# Existing psychological interventions addressing concerns about falling in older adults: a scoping review

**DOI:** 10.1093/ageing/afaf281

**Published:** 2025-10-19

**Authors:** Bianca Nicklen, Kim Delbaere, Toby Jack Ellmers

**Affiliations:** Department of Brain Sciences, Imperial College London, London W6 8RP, UK; School of Health Sciences, Faculty of Medicine and Health, University of New South Wales (UNSW) Sydney, Australia; Falls, Balance and Injury Research Centre, Neuroscience Research Australia, Randwick, New South Wales 2031, Australia; Department of Brain Sciences, Imperial College London, London W6 8RP, UK

**Keywords:** CaF, interventions, older adults, reviews, clinical practice

## Abstract

**Aims:**

Concerns about falling (CaF) are common in older adults. They are associated with increased falls and reduced quality of life. This scoping review aimed to (i) explore the psychological interventions that exist to address CaF in older adults and (ii) determine their feasibility and acceptability.

**Methods:**

The Arksey and O’Malley framework was used to identify all intervention studies that utilised a psychologically informed method to target CaF in older adults. Searches were conducted on five databases (Medline, CINAHL, Embase, Psychinfo and Scopus).

**Results:**

This review included 32 interventions (21 randomised controlled trials, five non-controlled trials, two non-randomised controlled trials and four case studies), comprising of 3674 participants. Thirteen different psychologically informed methods were used across interventions. Cognitive behavioural therapy (CBT) was the most common (n = 15), followed by exposure therapy (n = 4) and motivational interviewing (n = 3). Most interventions significantly reduced CaF. The median total dose across all interventions was 5 hours 40 minutes. CBT interventions tended to last the longest (median = 8 × 60-minute sessions). The shortest efficacious intervention involved a single ~20-minute session of motivational interviewing.

**Conclusion:**

Numerous psychologically informed techniques are currently used to address CaF—with CBT being the most common. However, the time and resource demands of many interventions may challenge their integration into clinical practice. Future work should explore the perspectives of those working in falls prevention services, and older adults themselves, to identify the most feasible and acceptable way to clinically manage CaF.

## Key Points

- The most common psychological approach for concerns about falling is cognitive behavioural therapy.
- This was followed by motivational interviewing and exposure therapy.
- The median total dose of existing interventions is 5 hours 40-minutes, with most interventions requiring extensive training for those delivering them.
- This scoping review included interventions in various settings (including community-dwelling, long-term care facilities and inpatients).
- Time and resource demands required for successful delivery raise questions about clinical feasibility of current interventions.

## Introduction

Concerns, or ‘fears’, about falling (CaF) are experienced by up to 85% of older adults [1]. CaF has been defined as ‘lasting feelings of dread and apprehension about situations that are believed to threaten or challenge balance’ (p.3 [2]). Whilst the term ‘fear of falling’ has been frequently used in the literature, the World Falls Guidelines underscore the advantages of utilising the term ‘concern’ [3]. This aims to move away from the emotionality associated with the construct of fear; with usage of the term ‘concern’ also helping to facilitate older adults’ willingness to disclose their experiences more openly [3]. Prevalence of CaF increases with age [1], and are higher in older adults who have fallen [1], as well as those who are frail or pre-frail [4]. Although some degree of concern can be adaptive—particularly when it reflects a realistic appraisal of fall risk [5–7]—CaF often leads to maladaptive outcomes. These include activity avoidance, social isolation, physical deconditioning and reduced mental wellbeing and overall quality of life [1, 8, 9]. A recent systematic review and meta-analysis also identified CaF as an independent predictor of future falls in community-dwelling older adults [10]. Consequently, the 2022 World Falls Guidelines [3] recommend that clinicians screen for CaF, and then implement targeted interventions when high levels of concern are detected [7].

CaF can be pervasive [11] and therefore require targeted intervention. In line with recent reviews [12], the World Falls Guidelines [3] recommended targeting CaF through holistic interventions that combine psychological with physical approaches (e.g. balance exercises, occupational therapy, etc). The most frequently used psychologically informed intervention to target CaF is cognitive behavioural therapy (CBT) [13–15]. CBT is a structured psychotherapeutic intervention, typically delivered over 8–20 sessions [16], designed to help people understand and modify problematic beliefs (‘cognitions’) and behaviours. A recent Cochrane review of randomised controlled trials (RCTs) identified probable evidence that CBT reduces CaF in older adults both immediately post-intervention and at 6-month follow-up, albeit with small effect sizes [17].

Despite this evidence, psychologically informed interventions for CaF do not appear to be commonplace within clinical practice. For instance, a survey of falls prevention practitioners in the UK and Ireland found that psychological interventions for CaF were only used by ~50% of respondents [18]. In this survey, a lack of time was the most frequently identified barrier to the effective clinical management of CaF. Indeed, the Cochrane review on CBT for CaF found that interventions ranged from 8 to 48 sessions [17]—durations that are likely unfeasible for most falls prevention services. Additionally, falls prevention practitioners require extensive training and support from specialist psychologists to effectively deliver formal CBT [19–21].

A more feasible approach could involve providing falls prevention practitioners with a toolbox of formal psychologically informed strategies (including, but not limited to, CBT-techniques) which they could incorporate alongside usual care. However, there is a lack of comprehensive knowledge regarding the existing psychological techniques used to address CaF. Current reviews have focused almost exclusively on the efficacy of CBT in RCTs, leaving gaps in understanding alternative methods, such as mindfulness, motivational interviewing and psycho-education, as well as their feasibility (i.e. number and length of sessions within the intervention) and acceptability (i.e. adherence/completion rate and recruitment rates into the intervention). Furthermore, there is little understanding surrounding the acceptability and feasibility of interventions between and within different settings (e.g. community-dwelling, long-term care facilities, and hospitals). This scoping review aimed to (i) explore the range of psychologically informed interventions across settings that are currently used to address CaF in older adults and (ii) examine the feasibility and acceptability of these strategies to inform future clinical implementation.

## Method

Scoping reviews allow a large breadth of research to be examined, including various study designs. The Arksey and O’Malley framework (and the Preferred Reporting Items for Systematic reviews and Meta-Analyses extension for Scoping Reviews [22]) were applied when conducting this review [23, 24] (see Appendix S2 in the supplementary materials).

### Identifying the research question

There is growing literature exploring the efficacy of psychological interventions on CaF in older adults [12, 15, 17, 25, 26]. However, previous reviews have focused primarily on the use of CBT interventions in RCTs, leaving knowledge gaps regarding alternative psychological techniques (e.g. mindfulness, motivational interviewing, psycho-education, etc.) and their feasibility and acceptability. Therefore, this scoping review aims to answer the following question: *What psychological interventions currently exist for targeting CaF, and how feasible and acceptable are these different strategies?*

### Identifying relevant studies

Studies were included if they investigated the effects of a psychologically informed intervention on CaF in older adults (aged 60 and above). Eligible studies reported quantitative CaF outcomes measurement, assessed through either a validated multi-item measurement (e.g. the Falls Efficacy Scale—International (FES-I) [27]) or a single-item assessment (e.g. ‘Are you worried about falling?’). Although a distinct construct to CaF [2, 28], studies assessing balance confidence (using, e.g. the original Falls Efficacy Scale (FES) [29] or the Activities-specific Balance Confidence (ABC) scale [30]) were included due to (i) its close relationship with CaF [2, 31] and (ii) the fact that seminal interventions designed to address ‘fear of falling’ (i.e. CaF) used balance confidence measures as their outcome measures [32]. Eligibility criteria were defined using the Population, Concept, Context framework [23, 33] (see Appendix S1 of the supplementary materials).

### Search strategy

Search terms were developed collaboratively by the authors and a university librarian including keywords: ‘older adults’, ‘aged’, ‘concerns about falling’, ‘fear of falling’, ‘psychological intervention’ and ‘behavioural therapy’. (See Appendix S3 of the supplementary materials for the full search terms). All searches were conducted in August 2023 (and updated in December 2024). Five databases were searched: Medline (Ovid) Embase (Ovid) Psychinfo (Ovid) CINAHL and Scopus. A further search for grey literature was conducted using Google Scholar and by screening reference lists of other related reviews and studies.

All searches were exported into Covidence [34], a web-based online collaboration software platform that removes duplicates and streamlines the production of reviews according to PRISMA [22, 35]. Using Covidence, the first (B.N.) and last author (T.J.E.) independently screened abstracts, selecting studies for full text review. Full texts were screened by the same two authors, with disagreements resolved through discussion or by consulting an independent third reviewer (K.D.).

### Charting the data

Charting the data was an iterative process. The first and last author (B.N and T.J.E) piloted data extraction on three studies, refining the categories for the final data extraction tables. Data were subsequently extracted by the first author (B.N.) and independently checked for accuracy by the last author (T.J.E.).

### Collating, summarising and reporting results

Extracted data were summarised in a table (Table 1), including study characteristics (i.e. authors, study design, country, sample description), psychological intervention, comparators, CaF and balance confidence outcome measures, adherence and recruitment rates, adverse events, follow-up period and the overall effectiveness of the intervention). An additional table detailed the psychological components of the intervention (i.e. psychological techniques used, specific techniques, implementation methods, etc; Table 2). Where relevant, authors were contacted to provide missing information.

**Table 1:** Outcomes from the included interventions*.*

**Author, year**	**Study Design**	**Country**	**Sample description**	**Psychological intervention**	**Comparator(s)**	**CaF/balance confidence outcome measure**	**Recruitment rate and adherence (and/or dropout) rate**	**Adverse events**	**Follow-up period**	**Key results**
Azizan & Justine, 2015	Non-randomised controlled trial	Malaysia	*N* = 62; Mean age = 63.5 (±3.9)**;** 56% female; community-dwelling; inclusion criteria not based on presence of CaF/low confidence; Baseline M-FES results: Control group = 5.8 (±0.7), Behavioural group = 5.7 (±0.6), Exercise only group = 5.7 (±0.7)	Behavioural therapy	1) Exercise only 2) ‘Talking’ control intervention, focused on discussing importance of physical activity and exercise	**Balance confidence:** 14-item M-FES (range = 0–10; higher scores = greater confidence)	**Recruitment rate:** Data not reported **Adherence rate:** Data not reported	Data not reported	12- and 24-weeks	**Pre-to-post changes** There was a significant increase in balance confidence both at 12-week post-intervention (M-FES mean increased by 31.2%, *P* < .001) and 24-week post-intervention (M-FES mean increased by 58.2%, *P* < .001) for the intervention group. **Comparison to control group** The intervention group had greater balance confidence at 12-week post intervention (*P* < .001) and 24-week post intervention (*P* < .001) when compared to the control group and exercise group.
Castro *et al*., 2021	Case study	UK	*N* = 1; Age = 78; female; community-dwelling; inclusion criteria based on ‘the abrupt presence of CaF’; Baseline FES-I = 28	Cognitive Physical Therapy	N/A	**CaF:** FES-I (range = 16–64; higher scores = greater CaF)	**Recruitment rate:** N/A (due to case study) **Adherence rate:** 100% adherence	Data not reported	1-month	**Pre-to-post changes** There was a reduction in CaF post-intervention (Short FES-I reduction of 14.0%). Due to being a case study, the significance is not reported.
Cuvelier *et al*., 2023	RCT	Switzerland	*N* = 32; Mean age = 85.6 (±5.8); 53% female; inpatients, geriatric rehabilitation wards; inclusion criteria not based on presence of CaF/low confidence; Baseline results for: perform-FES = 9.9 (±3.1); ABC = 23.4 (±8.4); GFFM = 44.7 (±12.5); FES-I = 32.3 (±12.5).	Hypnosis	Control group: usual care (included 2 weeks of intensive physiotherapy)	**Balance Confidence:** ABC (range = 0–100; higher scores = greater confidence) **CaF**: GFFM; FES-I (range = 16–64; higher scores = greater CaF); perform-FES (range = 7–28; higher scores = greater CaF)	**Recruitment rate:** 59% recruitment rate **Adherence rate:** 96% adherence to hypnosis sessions	No adverse events reported, including no falls.	Post-intervention only	**Pre-to-post changes** There was a non-significant reduction in CaF immediately post-intervention (FES-I mean reduction of 18.2%, *P* > .05). Pre- to post-intervention changes in other CaF outcomes were also non-significant (*P* > .05). **Comparison to control group** The group × time interaction was not significant (*P* > .05 for all CaF outcomes), meaning that the intervention group did not differ from the Control group post intervention.
Dorresteijn *et al*., 2011; 2013; 2016	RCT	Netherlands	*N* = 389; Mean age intervention group = 78.4 (±5.4) and 68% female; mean age control group = 78.3 (±5.3); 72% female; community-dwelling; participants recruited if they answered ‘sometimes’ (or more often) to the questions “Are you concerned about falling?” and 2) “Do you avoid certain activities due to concerns about falling?”; Baseline CaF results: FES-I, Control group = 35.5 (±9.4), Intervention group = 35.7 (±10.4); FES-IAB, Control group = 29.1 (±9.3), Intervention group = 29.1 (±9.7)	AMB-CBT (with additional motivational interviewing)	Control group: usual care	**CaF:** FES-I (range = 16–64; higher scores = greater CaF); FES-IAB (range = 16–64; higher scores = greater activity avoidance due to CaF)	**Recruitment rate:** 39% recruitment rate **Adherence rate:** 60% received at least 5 of the 7 sessions. Drop-out rate was higher in intervention group (31%) compared to control (17%). ‘Exposure *in vivo*’ exercise was only completed by half of the participants. 14% of intervention group withdrew due to ‘lack of interest’.	Data not reported	5- and 12-months	**Pre-to-post changes** There was a significant reduction in CaF at 5-month post-intervention (FES-I mean reduction of 11.1%, *P* < .001; FES-IAB mean reduction 9.9%) and at 12-month post-intervention (FES-I mean reduction of 10.4%, *P* < .001; FES-IAB mean reduction 9.3%). **Comparison to control group** CaF was significantly lower for the intervention compared to control group at both 5-month (FES-I, *P* < .001; FES-IAB, *P* = .001) and 12-month (FES-I, *P* < .001; FES-IAB, *P* = .001) post intervention, compared to the control group.
Finch *et al*., 2014; Parry *et al*., 2014, 2016	RCT	UK	*N* = 415; Mean age = 75.5 (±8.6); 70% female; community-dwelling; participants recruited if they scored >23 on the FES-I.	CBT	Control group: usual care	**CaF:** FES-I (range = 16–64; higher scores = greater CaF)	**Recruitment rate:** 66% recruitment rate **Adherence rate:** 32% dropped out from the intervention group and 21% from the control group	Eleven participants experienced a serious fall-related adverse event (n = 5 in the control and n = 6 in the intervention group)	Post-intervention; 6- and 12- months	**Overall changes** There was a significant reduction in CaF for the intervention group compared to the control group (mean difference in FES-I = −4.02, *P* < .001).
Faes *et al*., 2010	RCT	Netherlands	*N* = 33; Mean age = 78.3 years and 70% female; community-dwelling; inclusion criteria based on those having fallen within the last 6-months; baseline CaF not reported.	Psychological education programme	Control group: usual care	**CaF:** FES-I (range = 16–64; higher scores = greater CaF)	**Recruitment rate:** Data not reported **Adherence rate:** 88% completed the intervention (difference between groups is not reported)	Data not reported	Post-intervention, 3- and 6- months	**Overall changes** There was no significant between-group difference in pre-to-immediate-post intervention (3-months) change in FES-I scores (control = −3.62 SD = 8.59; intervention group = +1.78, SD = 8.51, *P* > .05). However, the pre-to-long-term (6-months) changes were significantly more positive for the control group (−1.06, SD = 8.73) compared to the intervention group who experienced *increased* CaF (+6.68, SD = 6.98, *P* = .038).
Giotakos *et al*., 2007	Non-controlled trial	Greece	*N* = 68; Mean age = 76.8 (± 5.2); 54% female; community-dwelling; inclusion criteria based on those who reported at ‘least some fear of falling and activity avoidance’; Baseline CaF results: All participants had high CaF (FES-I > 22) and low balance confidence (ABC = 47.6 (±25.7)).	VR-based Exposure Therapy	N/A	**CaF:** FES-I (range = 16–64; higher scores = greater CaF) **Balance confidence:** ABC (range = 0–100; higher scores = greater confidence)	**Recruitment rate:** Data not reported **Adherence rate:** Data not reported	Data not reported	6- and 12-months	**Pre-to-post changes** There was a reduction in CaF, with 97% of participants going from high CaF (> 22) measured by the FES-I to low CaF (< 22) at the 12-month follow-up post-intervention. Increases in balance confidence was observed at 6-month (ABC scores increased by 42.9%, *P* < .001) and 12-month post-intervention (ABC scores increased by 84.9%, *P* < .001) compared to baseline.
Healy *et al*., 2008	Non-controlled trial	USA	*N* = 335; mean age = 78.7 (± 8.3); 90% female; community dwelling; participants recruited on the basis on being ‘concerned about falling’; Baseline balance confidence (M-FES) = 3.3.	AMB-CBT	N/A	**Balance confidence:** 12-item M-FES (range = 1–4; higher scores = greater confidence)	**Recruitment rate:** Data not reported **Adherence rate:** 89% attended more than 5 out of 8 sessions	Data not reported	6- weeks, 6- months and 12-months	**Pre-to-post changes** There was an increase in balance confidence at both 6-months (M-FES score increased by 5.8%, *P* = .0005) and at 12-months (M-FES score increasing by 6.1%, *P* = .0013) post-intervention compared to baseline.
Huang *et al*., 2011	RCT	Taiwan	*N* = 186; aged >60 (mean ages not reported); 59% female; community-dwelling; inclusion criteria not based on presence of CaF/low confidence; Baseline CaF: GFFM, Control group = 38.4 (± 12.1), CBT group = 35.7 (±11.6), CBT + Tai Chi group = 35.8 (±9.8); Baseline balance confidence: FES, Control group = 90.4 (±16.8), CBT group = 88.8 (±17.4), CBT + Tai Chi group = 94.3 (±16.7)	CBT	Control group: usual care	**Balance confidence:** FES (range = 0–100; higher scores = greater confidence) **CaF:** GFFM (range = 15–75; higher scores = greater CaF)	**Recruitment rate:** 64% recruitment rate **Adherence rate:** 97% completed the trial	Data not reported	Post-intervention and 3-months	**Pre-to-post changes** There were small, non-significant increases in balance confidence post-intervention (FES score reduced by 1.5%, *P* > .05) and 3-months (FES score reduced by 2.3%, *P* > .05) for the intervention group compared to baseline. Similar patterns of non-significant improvement were observed for CaF (GFFM score decreased; post-intervention *n* = 10.2%, *P* > .05; 3-months = 9.3%, *P* > .05). **Comparison to control group** CaF, as measured by the GFFM, was significantly lower in the intervention group compared to control at both post-intervention (*P* < .05) and 3-months (*P* < .05). Balance confidence, as measured by the FES, did not significantly differ between intervention and control group at any timepoint (*P* > .05).
				CBT + Tai Chi			**Recruitment rate:** 64% recruitment rate **Adherence rate:** 90% completed the trial			**Pre-to-post changes** A significant reduction in CaF was observed at post-intervention (GFFM reduced in score by 9.9%, *P* < .05) and at 3-months (GFFM score reduced by 15.4%, *P* < .05) for the intervention group, compared to baseline. Non-significant improvements were observed for balance confidence (FES score increased, post-intervention *n* = 2.6%, *P* > .05; 3-months = 5.2%, *P* > .05). **Comparison to control group** CaF, as measured by the reduction in GFFM, was significantly lower in the intervention group compared to control at both post-intervention (*P* < .05) and 3-months (*P* < .05). Balance confidence was also similarly lower (*P* < .05).
Huang *et al*., 2016	RCT	Taiwan	*N* = 80; cognitive behavioural group mean age = 77.9 (±7.3), 59% female; cognitive behavioural group with exercise mean age = 79.1 (±7.0), 48% female; control group mean age = 81.3 (±5.3), 42%; nursing home residents; inclusion criteria not based on presence of CaF/low confidence; Baseline CaF: GFFM, Control group = 55.4 (±8.4), CBT group = 52.3 (±4.9), CBT + exercise group = 54.0 (±7.0); Baseline balance confidence: FES, Control group = 27.0 (±8.1), CBT group = 25.4 (±8.1), CBT + exercise group = 24.3 (±6.9).	CBT	Control group: usual care	**Balance confidence:** FES (range = 0–100; higher scores = greater confidence) **CaF:** GFFM (range = 15–75; higher scores = greater CaF)	**Recruitment rate:** 82% recruitment rate **Adherence rate:** 93% completed all the sessions	Data not reported	Post-intervention and 3-months	**Pre-to-post changes** Balance confidence did not significantly change following the intervention (*P* > .05). A significant reduction in CaF was observed at post-intervention (GFFM score lowered by 9.6%, *P* < .05) compared to baseline, but this was no longer significant at 3-months (mean value returning to baseline; *P* > .05). **Comparison to control group** CaF, as measured by the GFFM, was significantly lower in the intervention group compared to control at post-intervention (*P* < .05), but not at 3-months follow-up (*P* > .05). Balance confidence post-intervention was not significantly different compared to the control group.
				CBT + Exercise			**Recruitment rate:** 82% recruitment rate **Adherence rate:** 96% completed all sessions			**Pre-to-post changes** There was a significant increase in balance confidence at both post-intervention (FES score reduced by 8.6%, *P* < .05) and 3-months (FES score reduced by 24.6%, *P* < .05) for the intervention group compared to baseline. A significant reduction in CaF was observed at post-intervention (GFFM score reduced by 12.7%, *P* < .05), but not at 3-months (GFFM score reduced 8.3%, *P* > .05), compared to baseline. **Comparison to control group** CaF, as measured by the GFFM, was significantly lower in the intervention group compared to control at both post-intervention (*P* < .05) and 3-months (*P* < .05). Balance confidence, as measured by the FES, was similarly significantly higher both post-intervention and at 3-months compared to the control group (*P* < .05).
Juarbe & Bondoc, 2009	Case series	USA	*N* = 4; mean age 81.8 (±2.1); 75% female; community-dwelling; inclusion criteria not based on presence of CaF/low confidence; Baseline balance confidence results not reported.	Guided imagery and falls-based education	N/A	**Balance confidence:** 14-item M-FES (range = 0–10; higher scores = greater confidence)	**Recruitment rate:** N/A (as case series) **Adherence rate:** 100% completed the intervention	Data not reported	Post-intervention only	**Pre-to-post changes** Balance confidence increased post-intervention in 3 out of 4 participants (M-FES scores before and after the intervention are not reported).
Kampe *et al*., 2017; Pfeiffer *et al*., 2020	RCT	Germany	*N* = 115; mean age 82.5 (±6.8); 76% female; hip or pelvic fracture rehabilitation inpatients; inclusion criteria based on those who positively screened for CaF; Baseline CaF (Short FES-I), Control group =15.2 (±4.8), intervention group = 16.5 (±4.9)	CBT	Control group: usual care	**CaF:** Short FES-I (range = 7–28; higher scores = greater CaF)	**Recruitment rate:** 64% recruitment rate **Adherence rate:** 81% completed ≥5 sessions and had ≥3 telephone sessions after discharge, as well as having 1 home visit	Data not reported	1-month	**Pre-to-post changes** There was a significant reduction in CaF both directly post-intervention (Short FES-I mean reduction of 21.2%, *P* < .001) and at 1-month follow-up (Short FES-I mean reduction at 28.5%, *P* < .001) for the intervention group. **Comparison to control group** There was no significant difference in CaF between the intervention and control group at immediately post-intervention (*P* = .168); however, CaF (short FES-I) was significantly lower for the intervention group at 1-month post intervention (*P* = .025).
Kim *et al*., 2012	RCT	USA	*N* = 113; mean age, intervention group = 76.4 years (± 8.5) and 64% female; mean age, control group = 75.9 years (± 8.2) and 60% female; community-dwelling; inclusion criteria not based on presence of CaF/low confidence; Baseline CaF (short FES-I), Control group = 20.6 (±3.2), intervention group = 23.7 (±1.7).	Guided relaxation and imagery	Control group: instructed to listen to two of the same relaxation tracks as the intervention group, after listening to the relaxation track the participants listened to music of their choice for 5 minutes.	**CaF:** Short FES-I (range = 7–28; higher scores = greater CaF)	**Recruitment rate:** 95% recruitment rate **Adherence rate:** 83% completion in the intervention group; 84% completed in the control group	Data not reported	Post-intervention only	**Overall changes** Both groups showed a significant reduction in CaF (short FES-I scores; intervention group = −7.10, p = −0.002; control group = −2.09; *P* = .001); however, this improvement was significantly greater for the intervention group (*P* = .002).
Kim & Cho, 2022	RCT	Korea	*N* = 34; mean age, motor imagery training = 83.1 years (±5.2) and 60% female; mean age, VR-based exergame intervention group = 75.8 years (±10.2) and 58% female; mean age control group = 81.0 (±6.0) and 50% female; non-surgical inpatients; inclusion criteria not based on presence of CaF/low confidence; Baseline balance confidence (FES), Control group = 31.2 (±22.5), VR intervention group = 29.1 (±24.8), Motor imagery group = 31.2 (±24.3).	Motor Imagery Training	1) Control group: usual care; 2) VR-based exergame intervention group	**Balance confidence:** FES (range = 10–100; lower scores = greater confidence)	**Recruitment rate:** 97% recruitment rate **Adherence rate:** 100%	Data not reported	Post-intervention and 2-weeks	**Overall changes** There was no significant improvement in balance confidence (measured by a reduction in FES) for the intervention group either post-intervention or 2-week follow-up (*P* > .05). Likewise, no significant differences were observed between control and intervention group at any timepoints (*P* > .05).
Kiyoshi-Teo *et al*., 2019	RCT	USA	*N* = 67; mean age = 73.1 (±6.4); 3% female; general inpatients; participants were included if they were high fall risk as indicated by Morse Fall Scale (score ≥ 45); Baseline CaF (Short FES-I) = 17.8 (±6.6).	Motivational Interviewing	Control group: usual care	**CaF:** Short FES-I (range = 7–28; higher scores = greater CaF)	**Recruitment rate:** 69% recruitment rate **Adherence rate:** 100% completed the intervention	No adverse events were reported	2-days and 3-months	**Overall Changes** The intervention group exhibited significantly greater reductions in CaF (mean reduction in short FES-I = −2.00) compared to control group (mean change = +0.36, *P* = .009) 2-days post-intervention. These results remained in an adjusted analysis (*P* = .01). However, significant between-group differences were no longer observed at 3-months (adjusted *P* = .262).
Kiyoshi-Teo *et al*., 2024	RCT	USA	*N* = 185; mean age 80.1 (± 7.7); 68% female; community-dwelling; participants recruited based on high fall-risk (≥4 on the STEADI ‘Stay Independent’ questionnaire); Baseline CaF (Short FES-I), Control group = 13.8, Intervention group = 13.4	Motivational Interviewing	Control group: usual care	**CaF:** Short FES-I (range = 7–28; higher scores = greater CaF)	**Recruitment rate:** 53% recruitment rate **Adherence rate:** 68.3% completed the intervention	No adverse events were reported	Post-intervention and 6-months	**Overall changes** There was no significant pre-to-post change or between-group difference in CaF either post-intervention (short FESI-I, mean reduction: Intervention group = 0%; Control group = 4.3%; *P* = .46) or at 6-months (short FESI-I, mean reduction: Intervention group = 3.0%; Control group = 0%; *P* = .56).
Levy *et al*., 2016	RCT	France	*N* = 16; mean age, intervention group = 72.4 (±12.3) and 67% female; mean age, control group = 68.7 (±19.1) and 57% female; community-dwelling; participants recruited if they exhibited a ‘fear’ response during walking (e.g. sweating, heart racing) and activity avoidance; Baseline CaF (FFM), Control group = 39.0 (±7.7), Intervention group = 34.4 (±9.3)	VR-based Exposure therapy	Control group: waiting list, no extra treatment	**CaF:** FFM (range = 19–57; higher scores = greater CaF)	**Recruitment rate:** Data not reported **Adherence rate:** Completion rate not reported	Data not reported	Post-intervention only	**Overall change** The intervention group exhibited significantly greater reductions in CaF (mean reduction in FFM = −2.78) compared to the control group (mean change = +4.14, *P* = .007).
Lim *et al*., 2023	RCT	Australia	*N* = 50; 73.6 (±6.3) and 70% female; community-dwelling; participants recruited if they self-reported some CaF and/or poor balance confidence after answering the following screening questions ‘Are you concerned about falling during daily activities?’ and ‘How do you feel your balance is?’; Baseline CaF (IconFES), Control group = 52.9 (±12.0), Intervention group = 50.2 (±11.7); Baseline balance confidence (ABC), Control group = 83.1 (±14.2), Intervention group = 84.7 (±10.6).	Generalised CBT (i.e. not focused on CaF)	Control group: Paper-based health programme	**CaF:** IconFES (range = 30–120; higher scores = greater CaF) **Balance confidence:** ABC (range = 0–100; higher scores = greater confidence)	**Recruitment rate:** 82% recruitment rate **Adherence rate:** 76% completed all three intervention ‘modules’; 100% completed two out of three.	Data not reported	Post-intervention, 6-months, and 12-months	**Overall changes** There was a significant group × time interaction effect (*P* = .012) for CaF (iconFES)—with greater *reduction* in IconFES for the *control* group at 6- (−16.3%) and 12-months (−6.9%) compared to the intervention group (6-months = −3.4%; 12-months = +0.2%). There was no group × time interaction effect for balance confidence (ABC; *P* = .625).
Liu & Tsui 2014	RCT	China	*N* = 122; mean age, intervention group = 74.5 (±7.5 and 88% female; mean age, control group = 74.5 (±7.1) and 86% female; community-dwelling; participants recruited if they scored >23 on the FES-I; Baseline CaF (FES-I), Control group = 29.7 (±6.6), Intervention group = 30.0 (±5.5).	CBT	Control group: Tai Chi	**CaF:** FES-I (range = 16–64; higher scores = greater CaF)	**Recruitment rate:** 67% recruitment rate **Adherence rate:** 77% completed more than 50% of the training sessions	Data not reported	Post-intervention and 2-months	**Pre-to-post changes** There was significant reduction in CaF both directly post-intervention (FES-I mean difference of 15.1%, *P* < .001) and 2-months post-intervention (FES-I mean difference of 21.4%, *P* < .001) for the intervention group. However, the control group saw comparable (and statistically significant) reductions in CaF post intervention (19.5%; *P* < .001) and 2-months post interventions (22.7%; *P* < .001). **Comparison to control group** Pre-to-post changes in CaF were not significantly different between the intervention and control group (time × group interaction: *P* = .78).
Mansdorf *et al*., 2009	Non-controlled trial	USA	*N* = 26; mean age 80.7; 62% female; long-term care facility; participants recruited based on being deemed at high risk for falling; Baseline CaF results not reported.	CBT	N/A	**CaF:** FES-I (range = 16–64; higher scores = greater CaF)	**Recruitment rate:** Data not reported **Adherence rate:** Data not reported	Data not reported	Post-intervention only	**Overall changes** No formal statistics presented; but 47.0% showed slight worsening of CaF (mean increase in FES-I of 11.4%) while 32.5% reported a reduction (mean difference 28.2%). Finally, no change was observed in 17.7%.
O’Halloran *et al*., 2016	RCT	Australia	*N* = 30; mean age 82.6 (± 5.2); 84% female; community-dwelling; inclusion criteria not based on presence of CaF/low confidence; Baseline balance confidence results: M-FES for the control group = 7.4 (±1.7); M-FES for the intervention group = 8.2 (±2.3)	Motivational Interviewing	Control group: usual care	**Balance confidence:** 14-item M-FES (range = 0–10; higher scores = greater confidence)	**Recruitment rate:** 46% recruitment rate **Adherence rate:** 92% completed 100% of sessions; 8% completed 7 out of 8 sessions.	No adverse events were reported	1-week	**Overall changes** The intervention group exhibited a slight improvement in balance confidence (M-FES increase of +2.4%) versus a decrease observed in the control group (−9.5%), with this difference in change score statistically significant (*P* = .007).
Reinsch *et al*., 1992	RCT	USA	*N* = 230; Mean age, cognitive-behavioural group = 74.9 (±5.8) and 77% female; mean age, cognitive-behavioural + exercise group = 73.9 (±6.7) and 71% female; mean age exercise control group = 73.4 (±7.6) and 86%; mean age, discussion control group 75.7 (±7.2) 92% female; community-dwelling; inclusion criteria not based on presence of CaF/low confidence; Baseline CaF (5-point scale), Controls (discussion only) = 1.3 (±0.9), Controls (exercise only) = 1.4 (±0.9), Relaxation intervention *n* = 1.4 (±0.7), Relaxation intervention + exercise = 2.3 (±0.7)	Relaxation training and falls-based education	Control groups: 1) discussion only, 2) exercise only (‘stand-up/step-up’ programme)	**CaF:** 5-point scale (1 = not at all worried about falling to 5 = extremely worried)	**Recruitment rate:** Data not reported **Adherence rate:** 73% completed at least 66% of the relaxation training and falls-based education intervention; 85% completed at least 66% the relaxation training and falls-based education + exercise intervention	Data not reported	Post-intervention only	**Overall changes** There was no significant pre-to-post change in CaF for either intervention groups (+14.3% for relaxation only; 0% for relaxation + exercise; *P* > .05)—nor any post-intervention differences between intervention or control groups (*P* > .05).
				Relaxation training and falls-based education + exercise						
Scheffers-Barnhoom *et al*., 2017; 2019; 2021	RCT	Netherlands	*N* = 77; mean age = 82.5 (± 7.6); 79% female; inpatient rehabilitation; participants were recruited if they answered ‘sometimes’ to ‘very often’ to the question: ‘Are you concerned to fall?’; Baseline CaF (FES-I) = 34.2 (±10.6)	CBT (with additional motivational interviewing)	Control group: usual care	**CaF:** FES-I (range = 16–64; higher scores = greater CaF)	**Recruitment rate:** 72% recruitment rate **Adherence rate:** 92% completed the intervention. (Note, the decision of what components to deliver to the participants was individualised, therefore, guided exposure delivered to 97% of participants and cognitive restructuring to 72%)	More falls were reported in the control group	Post-intervention, 3- and 6-months	**Overall changes** There was no significant change in FES-I scores from baseline for either group at any timepoint (*P* = .84). Interestingly, there was a trend towards greater reduction in CaF at discharge for the control group with a reduction in FES-I scores (−21.5%) versus the intervention group (−3.2%), but this difference was not statistically significant (*P* = .13).
Sibley, 2024	Case study	UK	*N* = 1; 77 years old; female; supported living; inclusion criteria based on having high CaF; Baseline CaF (FES-I) = 60.	CBT	N/A	**CaF:** FES-I (range = 16–64; higher scores = greater CaF)	**Recruitment rate:** N/A (as case study) **Adherence rate:** 100% completed	Data not reported	Post-intervention only	**Pre-to-post changes** There was a reduction in FES-I scores post-intervention, with a reduction of 63.8% from baseline (score changed from 58/64 to 26/64), indicating a large reduction in CaF
Tennstedt *et al*., 1998	RCT	USA	*N* = 434; mean age = 77.8 (±7.7); 90% female; community-dwelling; participants were included based on self-reported restriction in activity due to CaF; Baseline balance confidence (M-FES) = 2.6 (±0.7).	AMB-CBT	Control group: Single 2-hour group session about incidence and risk factors of falls (CaF was not discussed)	**Balance confidence:** 12-item M-FES (range = 1–4, higher scores = greater confidence)	**Recruitment rate:** Data not reported **Adherence rate:** 63% completed over 5 of the 8 sessions; 20% completed 1–4 sessions; 16% attended zero sessions	Data not reported	6-weeks, 6-months and 12-months	**Overall changes** There was a significantly greater improvement in balance confidence for those that completed the intervention (mean increase in confidence = +0.15) compared to the control group (mean reduction in confidence = −0.04, *P* < .01) at 6-week follow-up. Similar patterns were observed at 12-months (mean increase, intervention *n* = +0.09; mean reduction, control = −0.12, *P* < .01). There were no between-group differences at 6-months (*P* > .05).
Thiamwong *et al*., 2020	RCT	USA	*N* = 41; mean age, intervention group = 75.5 (±5.4) and 74% female; mean age, control group = 78.3 (±9.1) and 59% female; community dwelling; inclusion criteria not based on presence of CaF/low confidence; Baseline CaF (Short FES-I), Control group = 13.0 (±6.8), Intervention group = 10.7 (±4.7).	Visual physio-feedback and cognitive reframing	Control group: review falls prevention brochures for 60-minutes per week, for 8-weeks	**CaF:** Short FES-I (range = 7–28; higher scores = greater CaF)	**Recruitment rate:** Data not reported **Adherence rate:** 100% completed	Data not reported	Post-intervention only	**Overall changes** There was no significant change in CaF (short FES-I) post-intervention for the intervention group (−5.4%, *P* = .343). However, the control group exhibited a significant reduction in CaF post-intervention (with a reduction in short FES-I by −5.9%, *P* = .018).
Tsai *et al*., 2023	Non-randomised controlled study	Taiwan	*N* = 72; mean age, intervention group = 81.9 (±5.4) and 44% female; mean age, control group = 83.1 (±5.4) and 39% female; long-term care facility; inclusion criteria not based on presence of CaF/low confidence; Baseline CaF (Short FES-I), Control group = 15.6 (±7.2), Intervention group = 17.3 (±7.6).	Mindfulness	Control group: usual care	**CaF:** Short FES-I (range = 7–28; higher scores = greater CaF)	**Recruitment rate:** Data not reported **Adherence rate:** 100% completed all 8 sessions	Data not reported	Post-intervention, 3-months	**Pre-to-post changes** There was a significant reduction in CaF (short FES-I) both at directly post-intervention (mean reduction *n* = −40.5%, *P* < .001) and 3-months post-intervention (mean reduction *n* = −41.5%, *P* < .001) for the intervention group. **Comparison to control group** The reduction in CaF was significantly greater for the intervention compared to the control group both post-intervention (*P* < .001) and 3-month post intervention (*P* < .001).
Tsujishita *et al*., 2020	Case study	Japan	*N* = 1; 87 years old; community-dwelling; the participant was recruited based on having had a previous fall; Baseline balance confidence measured (M-FES) = 108.	VR-based exposure	N/A	**Balance confidence:** 14-item M-FES (range = 36–140; higher scores = greater confidence)	**Recruitment rate:** N/A (as case study) **Adherence rate:** 100% completed	Data not reported	Post-intervention only	**Pre-to-post changes** There was a slight increase in balance confidence post-intervention (M-FES increase of 5.6%) in the case study.
Van Haastregt *et al*., 2007; Zijlstra *et al*., 2009; Zijlstra *et al*., 2011	RCT	Netherlands	*N* = 540; mean age, intervention group = 77.8 (±4.6) and 71% female; mean age, control group = 78.0 (±5.0) and 73% female; community-dwelling; participants were included based on self-reported restriction in activity due to CaF; Baseline CaF (FES-I), Control group = 30.0 (±10.2), Intervention group = 28.5 (±9.6).	AMB-CBT	Control group: usual care	**CaF:** FES-I (range = 16–64; higher scores = greater CaF)	**Recruitment rate:** 40% recruitment rate **Adherence rate:** 94% completed the trial, of which 62% completed more than 6 out of 8 sessions.	No adverse events were reported	Post-intervention, 6- and 12-months	**Pre-to-post changes** There was a significant reduction in CaF (FES-I) for the intervention group post-intervention (mean reduction *n* = −15.0%, *P* < .001), and at 6- (−16.3%, *P* < .001) and 12-months (−12.3%, *P* < .001), compared to baseline. **Comparison to control group** The reduction in CaF was significantly larger for the intervention group compared to control group both directly post-intervention (*P* = .02) and 6-months post-intervention (*P* = .002); but this difference was no longer statistically significant at 12-months post intervention (*P* = .07).
Wetherell *et al*., 2016	Non-controlled trial	USA	*N* = 8; mean age = 80.7 (±8.0); 90% female; community-dwelling; participants were included if the participants’ CaF was ‘excessive’ relative to their physical function; baseline CaF results not reported.	CBT	N/A	**CaF:** Short FES-I (range = 7–28; higher scores = greater CaF)	**Recruitment rate:** Data not reported **Adherence rate:** Two participants were unable to participate: one fell and broke their hip, and another was too disabled to participate. Of the other 8, 100% completed all 8 sessions	During the trial, 5 participants reported a single fall, 1 participant reported 2 falls.	Post-intervention only	**Overall changes** There was a significant reduction in CaF (mean reduction in short FES-I = −6.1) post-intervention (*P* = .012).
Wetherell *et al*., 2018	RCT	USA	*N* = 42; mean age = 77.9 (±7.3); 74% female; community-dwelling; participants were included if they had high CaF (scoring >27 on FES-I); Baseline CaF: FES-I for the control group = 39.1; FES-I for the intervention group = 41.0.	CBT	Control group: Fall prevention education (8 weekly 1-hour sessions)	**CaF:** FES-I (range = 16–64; higher scores = greater CaF)	**Recruitment rate:** 47% recruitment rate **Adherence rate:** 91% completed all sessions; two people dropped out, one of which dropped out due to ‘increased anxiety’	Data not reported	Post-intervention, 8-weeks, 3- and 6- months	**Overall changes** The intervention group exhibited significantly greater reductions in CaF (FES-I) post-intervention (*P* = .02), and at 8-weeks post-intervention (*P* = .03). However, the differences were lost during follow-up, with the between-group differences no longer significant at 3- (*P* = .06) or 6-months (*P* = .46).
Zijlstra *et al*., 2013	Non-controlled study	Netherlands	*N* = 125; mean age = 77.7 (±6.2); 70% female; community-dwelling; inclusion criteria not based on presence of CaF/low confidence; Baseline CaF (Short FES-I) = 13.7 (±4.4).	AMB-CBT	N/A	**CaF:** Short FES-I (range = 7–28; higher scores = greater CaF)	**Recruitment rate:** 95% recruitment rate **Adherence rate:** 93% completed the intervention	Data not reported	Post-intervention, 2-months	**Pre-to-post changes** There was a significant reduction in CaF directly post-intervention (Short FES-I mean reduction of 5.8%, *P* = .006) and 2-months post-intervention (Short FES-I mean reduction of 5.8%, *P* = .047).

ABC = Activity Balance Confidence Scale; AMB-CBT = A Matter of Balance—Cognitive behavioural Therapy; CaF = Concerns about Falling; CBT = Cognitive Behavioural Therapy; FES = Falls Efficacy Scale; FES-I = Falls Efficacy Scale—International; FES-IAB = Falls Efficacy Scale—International Avoidance Behaviour; FFM = Fear of Falling Measure; GFFM = Geriatric Fear of Falling Measure; Icon FES = Icon Falls Efficacy Scale; M-FES = Modified Falls Efficacy Scale; Perform-FES = Perform Falls Efficacy Scale; Short FES-I = Short Falls Efficacy Scale—International; RCT = Randomised Controlled Trial; VR = Virtual Reality.

**Table 2:** Details of the psychological method/s used in the included intervention.

Study	Intervention design				
	Psychological technique	Description	Duration	Delivered: by & setting	Information on training provided for implementers
Azizan & Justine 2015	Behavioural therapy	Topics covered in the weekly sessions included: Discussions around changes in behaviours, the risks of falls, and how inactivity can affect an individuals’ social activities; Conversations around self-confidence; Strategies to form new self-image and handle CaF; Group counselling on positive reinforcement; Discussions on how to engage in exercise and physical activity.	10 sessions of 30 minutes (2x per week) delivered over 5 weeks, directly after the exercise component of the study.	Delivered face-to-face; Physiotherapist; Sessions in week 1, 2, 4 and 5 delivered in groups, and session in week 3 delivered 1–1; All sessions were delivered in community centres.	Data not reported
Castro *et al*., 2021	Cognitive Physical Therapy	Gait was retrained while attention was diverted away from conscious motor control (via auditory cues and cognitive distractors). The patient was encouraged to use relaxation techniques while walking, and to use realistic thinking strategies to explore and manage underlying CaF. Focus was placed on rationalising the patient’s perceived postural instability to avoid catastrophisation. The patient was encouraged to practice what was learned from each session at home.	3 × 30-minute sessions (1 × month); follow-up sessions occurred once a month over a 3-month period.	Delivered face-to-face; Audiologist; 1–1; Delivered in hospital setting.	The audiologist was previously trained in CBT.
Cuvelier *et al*., 2023	Hypnosis	During the participants’ hypnosis sessions, they were asked to imagine walking and/or to focus on how they felt when walking. The aim was to have the participant focus on their sensations and memories related to walking, instead of CaF. Focus was placed on specific parts of the experience unrelated to CaF, e.g. getting the participant to think of smells and sounds, etc.	4 sessions of 30 minutes (2x per week) delivered over 2 weeks.	Delivered face-to-face; Physician; 1–1; Delivered in hospital setting.	The physician was previously certified and trained in medical hypnosis.
Dorresteijn *et al*., 2011; 2013; 2016	AMB-CBT	The intervention used the home version of AMB, developed by Tennstedt *et al*. (1998) which was translated and adapted into Dutch. Please see the Tennstedt *et al*. (1998) entry in this table for full description of the intervention. A few key adaptations were made including reducing the sessions to 7 (from 8). One session also involved participants being guided to perform a daily task they were concerned about (‘exposure in-vivo’). Motivational interviewing was also applied to help participants overcome their beliefs and behaviours towards activity restriction and concerns about falls.	7 sessions (3 home visits (60, 60 and 75 minutes) and 4 telephone appointments (35 minutes each)). The first 4 sessions occurred weekly and the last 3 occurred every 2 weeks.	Delivered face-to-face and over the telephone; Community nurses; 1–1; Delivered in older adults’ homes and over the telephone.	Facilitators received 2 days of training consisting of studying the manual and workshops held by professionals in motivational interviewing, behaviour change and exposure in-vivo.
Finch *et al*., 2014; Parry *et al*., 2014; 2016	CBT	The sessions consisted of various CBT techniques that were applied where needed, based on the individual’s beliefs and behaviours towards CaF. Techniques included: cognitive restructuring; strategies to challenge unhelpful or maladaptive beliefs; graded exposure activities; behavioural management strategies for pain, fatigue and low mood, and; goal setting.	8 weekly 45-minute sessions, and 1 booster session 6 months later.	Delivered face-to- face; Healthcare assistant; 1–1; Delivered in older adults’ homes.	Healthcare assistants received 5 days training on CBT and skills of engagement with patients.
Faes *et al*., 2010	Psychological education programme	Topics covered included: Goal setting; how CaF can create a vicious cycle by reducing physical activity, then recognising the feelings and emotions this causes, and using cognitive restructuring to help reduce the concerns; the ‘Stop-think-go method’, to help the participant carefully think and plan their tasks before completing them; problem-focused coping strategies to help increase levels of self-efficacy opposed to avoidance-oriented coping strategies. Broad topics related to falls were also covered, including: discussions to help increase understanding about how falls happen and methods to prevent them; the use of safety and walking aids; how to get up after a fall; how to ask for assistance.	10 sessions of 2-hours (2x per week) for 5 weeks; followed by a single 2-hour booster session 6 weeks after the final session.	Delivered face-to-face; Geriatric psychologist and physiotherapist; Group sessions of 5 pairs of patients and their caregivers; Location information not reported.	Delivered by a qualified geriatric psychologist.
Giotakos *et al*., 2007	VR-based exposure therapy	Participants were exposed to VR environments while walking on a treadmill. These environments were designed to represent threatening/challenging situations where a fall might be likely to occur (e.g. slippery floor/icy floor). Participants performed challenging balance tasks while walking (e.g. stepping over virtual obstacles/trip hazards). A dual-task element was introduced in the final week, whereby participants completed the task while also counting aloud or carrying a glass of water.	9 sessions of 10 minutes (3x per week) for 3 weeks; 1 booster session at 6 months.	Delivered face-to-face; Physiotherapist; Location and setting not reported.	Data not reported
Healy *et al*., 2008	AMB-CBT	The intervention used AMB, developed by Tennstedt *et al*. (1998). Please see the Tennstedt *et al*. (1998) entry in this table for full description of the intervention.	8 sessions of 2-hours (2x per week) over 4 weeks.	Delivered face-to-face; Volunteer lay leader; Group sessions; Delivered in community settings.	The volunteer lay leaders received 2 days training.
Huang *et al*., 2011	CBT	The intervention was focused on restructuring misconceptions around fall risk and helping to make CaF be perceived as controllable. Namely, focus was placed on countering maladaptive cognitions and beliefs that falling is an inevitable part of ageing, and helping participants develop behavioural strategies to prevent them from curtailing their activities due to CaF.	8 weekly 60–90-minute sessions.	Delivered face-to-face; Geriatric nurse; Group sessions (8–12 participants); Location not reported.	Data not reported
Huang *et al*., 2016	CBT	A condensed, shortened version of the same intervention from Huang *et al*., 2011 was used.	8 weekly 20–25-minute sessions.	Delivered face-to-face; Geriatric nurse; Group sessions (6–8 participants); Delivered in nursing homes.	Data not reported
Juarbe & Bondoc, 2009	Guided imagery and falls-based education	The sessions consisted of an educational component followed by guided imagery. The educational component consisted of discussions on how to improve awareness about causes of falls (e.g. environmental factors, activity-based factors). There were also discussions around how to increase confidence, and education on the physiological and psychological characteristics that are associated with CaF.	6 weekly 35–45-minute sessions, and a self-administered home programme.	Delivered face-to-face; A facilitator; Group sessions; delivered within an older adult centre and self-directed sessions conducted in the participants’ homes.	Data not reported
		The guided imagery involved a 16-minute recording. The format followed deep breathing and muscle relaxation techniques. The listeners were then guided through a morning walk through their neighbourhood. They were asked to be mindful of their own pace and of their environment, paying attention to their own physiological responses to the guided walk (e.g. changes in breathing or muscle tension). The home programme was self-administered and included the same guided imagery audio recording, along with a recap of the covered educational information.			
Kampe *et al*., 2017; Pfeiffer *et al*., 2020	CBT	This multicomponent intervention used various CBT-based techniques, including: Addressing cognitions and behaviours around CaF, using the ABC model (A = adversity, B = beliefs, C = consequences). This involved gradual exposure to situations that triggered CaF while utilising progressive muscle relaxation techniques; Developing adaptive strategies and behaviours to overcome threatening scenarios using structured problem-solving; Identification of fall risks and hazards; Setting of meaningful physical activity and mobility-based goals both within the in-patient rehabilitation setting and following discharge (including identification of barriers to these goals and strategies to address these).	8 sessions of 30–60-minutes, delivered across 3–5 weeks (during in-patient stay), followed by 1 home visit and 4 telephone calls post-discharge.	Delivered face-to-face and over the telephone; Physiotherapists or sports scientists; 1–1 sessions; First 8 sessions delivered in geriatric inpatient rehabilitation; 1 home visit and 4 telephone calls post-discharge.	Those delivering the intervention received training in CBT and relaxation techniques by a clinical psychologist. The clinical psychologist then supervised the physiotherapists and sport scientist throughout the intervention.
Kim *et al*., 2012	Guided relaxation and imagery	The intervention consisted of a CD featuring guided relaxation and imagery exercises. The two guided relaxation exercise tracks consisted of progressive muscle relaxation and deep breathing. This was followed by 11 guided imagery exercises tracks consisting of short (4–6 minutes long) imagery scenarios that progressively became more threatening/challenging, starting with activities around the house and progressing to walking on an icy road.	Instructed to listen to the CD for 15 minutes twice per week, for 6 weeks.	Self-administered; 12 individual pre-recorded CD tracks.	The researchers who recorded the audio CD were trained in relaxation and guided imagery.
Kim & Cho, 2022	Motor imagery training	The intervention was delivered in a quiet, dark room. The participants were asked to sit in a chair with armrests and to close their eyes while listening to the therapists’ instructions. The intervention was divided into two components: the motor- and the visual-sensory imagery training. For the motor-sensory imagery training the participants were asked to feel the pressure in the soles of the feet and how it felt to shift their weight between their feet. For the visual-sensory imagery, the participants were asked to imagine and observe their body movements from the perspective of a third person.	18 sessions of 20-minutes (3x per week), over 6 weeks.	Delivered face-to-face; Researchers; Group number not specified, in a quiet location to promote participant concentration.	Data not reported
Kiyoshi-Teo *et al*., 2019	Motivational Interviewing	Motivational Interventional approaches such as open-ended questions to evoke behaviour change, affirmations, and reflections on fall prevention. Permission was sought from the participants before offering information or asking questions. Questions used included: ‘What does fall prevention mean to you?’ and ‘What are one or two things that may be useful or meaningful to you for us to discuss about fall prevention?’ The intention was to let the participants identify what specific behaviours could help reduce falls (e.g. asking for help, using walking aids) to help empower and build confidence in the participants.	1 × 20-minute session.	Delivered face-to-face; Registered nurse; 1–1 individual session delivered in the hospital.	The researchers who delivered the motivational interviewing interventions had received ≥40 hours training on motivational interviewing.
Kiyoshi-Teo *et al*., 2024	Motivational Interviewing	The same intervention from Kiyoshi-Teo *et al*., 2019 is used, but the intervention was delivered outside of the hospital setting, over the telephone or via video calls.	8 × 15—30-minute sessions delivered over a 6-month period.	Delivered over the telephone or online; Registered nurse or psychologists; 1–1 individual sessions delivered by telephone or video calls.	The researchers who delivered the motivational interviewing interventions had received ≥40 hours training on motivational interviewing.
Levy *et al*., 2016	VR-based Exposure Therapy	Subjects were asked to navigate through VR environments while seated using a wireless mouse. There were four different virtual environments of increasing threat/challenge to balance: Environment 1 featured a cityscape with level ground; Environment 2 involved walking through a castle with narrow corridors and a high step; Environment 3 featured uneven ground of varying heights; Environment 4 consisted of a steep flight of steps.	12 weekly sessions of 40-minutes.	Delivered face-to-face; Physiotherapist; 1–1 individual sessions; Location not specified.	Data not reported
Lim *et al*., 2023	Generalised CBT (i.e. not focused on CaF)	Participants completed three online CBT modules (not specific to CaF) which included interactive activities. These focused on: Identifying negative thoughts and behaviours; Identifying normal compared to excessive worry and providing therapeutic advice on how to overcome excessive worry; Providing a structured approach to help participants solve any stressful problems experienced in a healthy manner.	6 weekly 10-minute online modules, plus practice in their own time.	Self-administered; Online programme delivered via an app.	The intervention was delivered online, and not by an implementer.
Liu & Tsui, 2014	CBT	Sessions focused: on restructuring misconceptions around CaF and promoting the belief that it is controllable; realistic goal setting to promote physical activity; cognitive restructuring around physical activity.	8 weekly sessions of 60–90-minutes.	Delivered face-to-face; Researchers; Group sessions (10–11 participants) delivered in community centres.	The researchers delivering the intervention received 8 hours of training conducted by a licensed cognitive behavioural therapist.
Mansdorf *et al*., 2009	CBT	The intervention focused on modifying maladaptive behaviours and cognitions regarding falling. Sessions consisted of interactive activities designed to teach the participant how to identify unhelpful behaviours. For instance, the participant viewed different animated skits, each of which featured a specific ‘problem’ related to fall risk. The solution to the problem was left open and a psychologist would aid the	12 weekly sessions of 35–45 minutes (more sessions could be delivered if the psychologist ‘felt this was necessary’; information on average number of extra sessions not reported)	Delivered face-to-face; Psychologist; 1–1; Delivered in the long-term care facilities.	The psychologists all received individualised training for the intervention. Time spent on training was not mentioned.
		participant to correctly solve the problem being asked. The exercises focused on different behavioural factors associated with falls risk, e.g. learning to ask for help, learning to assess and scan an environment for hazards. Each exercise was practiced and reviewed until the individual understood the behavioural factor being assessed. Various cognitive techniques were also involved in the sessions, including cognitive restructuring, self-instruction, and behavioural rehearsal.			
O’Halloran *et al*., 2016	Motivational Interviewing	Telephone-based motivational interviewing was conducted alongside usual care following discharge post hip fracture. Motivational interviewing was conducted by working collaboratively to assist the participant in forming their own arguments for change. This aimed to address participants’ ambivalence about engaging in physical activity, as well as improving their low balance confidence and reducing their CaF.	8 weekly 30-minute sessions.	Delivered over the telephone; Physiotherapist; 1–1, telephone-based sessions.	The physiotherapist delivering the motivational interviewing completed a 2-day workshop, online training, and received 1–1 coaching from an experienced motivational interviewing practitioner
Reinsch *et al*., 1992	Relaxation training and falls-based education	The sessions consisted of a relaxation component to lower tension and CaF, health and safety education to prevent falls, and a video game that is designed to improve reaction time.	52 weekly 60-minute sessions.	Delivered face-to-face; Data not reported on who delivered the session; Group classes; Delivered in an older adult centre.	Data not reported
Scheffers-Barnhoom *et al*., 2017; 2019; 2021	CBT	The intervention consisted of various elements, including: Guided exposure to feared activities. This involved developing a 6-step ‘fear ladder’ where each step is a goal towards their ultimate personalised functional goal. Each step increases the exposure gradually and is repeated until the fear of each step subsides, before moving to the next step; Cognitive restructuring, looking at current limiting thoughts and feelings towards physical activity; Psychoeducation was delivered throughout the intervention, to help apply rationale to situations that might occur in their daily activities or that happen during the intervention; Relapse prevention; Motivational interviewing was additionally conducted throughout to help evoke behaviour change related to physical activity. This involved exploring the participants’ intrinsic and extrinsic motivations to help overcome ambivalence towards change.	On average, 30.7 physiotherapy sessions were delivered. Due to the nature of the intervention, the specific durations and times of the psychological component are not specified. However, the mean number of guided exposures delivered was 18.9 sessions per participant; the mean number of cognitive restructuring sessions was 3.5; and the mean number of psychoeducation sessions was 1.9.	Delivered face-to-face; Physiotherapists; 1–1 sessions; Delivered in a nursing home.	The physiotherapists who delivered the intervention had ‘prior training’ (details not specified). They were provided with additional support from a psychologist throughout the intervention, as needed (who could also provide additional treatment for more complex psychiatric problems). Nursing staff were trained and instructed on how to deliver guided exposure. Motivational interviewing training consisted of two training days (4 hours each).
Sibley, 2024	Graded exposure	The graded exposure intervention was informed by Parry *et al*., 2016’s CBT framework. The 12 structured sessions included: setting goals, creating a hierarchy for exposure, identifying maladaptive thoughts and linking them to behaviours, practicing anxiety-reducing strategies, addressing any barriers to exposure and, finally, how to prevent a relapse.	12 sessions (once per week), duration of sessions was not reported.	Delivered face-to-face; Trainee psychologist; 1–1 sessions; Data not reported on where the intervention was delivered.	Data not reported
Tennstedt *et al*., 1998	AMB-CBT	The original ‘A Matter of Balance’ (AMB) programme. The cognitive restructuring approach was used to instil adaptive beliefs towards the participants’ perceived control and confidence around preventing a fall. Sessions focused on identifying and changing maladaptive beliefs and cognitions. This was reinforced using didactic material involving information on the risk of falling and skill training in falls prevention with what to do if someone falls. Participants were also taught assertive methods, to encourage them to have conversations about CaF with caregivers and healthcare professionals. Finally, behavioural contracts and/or goal setting were implemented. This was centred around the specific behaviour an individual wanted to change and/or goal they wanted to achieve. Additional components involved education surrounding the physical and psychological costs of inactivity, and the benefits of strength and balance exercise for falls prevention. This is delivered alongside exercise and balance training.	8 sessions of 2-hours (2x per week) for 4 weeks.	Delivered face-to-face; Trained facilitators; Group session; Delivered in older adult housing buildings.	Data not reported
Thiamwong *et al*., 2020	Visual physio-feedback and cognitive reframing	Participants completed the Short FES-I to assess an individual’s perceived fall-risk, as well as an objective test of physiological fall-risk (the BTrackS Balance Test). The results enabled participants to be placed into an appraisal matrix, based on their CaF: Irrational: high perceived falls risk, low physiological falls risk; Rational: low perceived fall risk, low physiological falls risk; Congruent: high perceived falls risk, high physiological falls risk; Incongruent: low perceived falls risk, high physiological falls risk. Participants received visual feedback on their balance performance. The fall risk appraisal matrix was used by the researcher with the participants to help guide the cognitive restructuring, goal setting and tailor the treatment activities for the participants.	Cognitive restructuring session details not reported	Delivered face-to-face; Researcher; 1–1 and group sessions; Community sites.	Data not reported
Tsai *et al*., 2023	Mindfulness	The mindfulness intervention consisted of exercises using ‘outer wisdom’, which is the knowledge of motion, and ‘inner wisdom’, which is where the individual practices mindful behaviour to help perceive their own body’s responses and regulations of movement. For outer wisdom, the participants were given a set of exercise cards to be completed in each session. The card contained specific information on a particular movement and exercise to be performed (e.g. ankle or torso rotation), as well as the muscles used to achieve them. Instructions were provided to help participants focus on contracting and relaxing specific muscles, and practice specific body mechanics/transitions (e.g. lying to sitting, sitting to standing, standing to walking). For inner wisdom, a mindfulness teacher taught the participants how to conduct and practice body scans and breathing meditations and develop mindfulness beliefs (e.g. self-acceptance and forgiveness).	8 weekly 100-minute sessions; homework to use the weekly cards to help with body scans and meditations (~30 minutes per day).	Delivered face-to-face and also self-administered; A mindfulness teacher; Group sessions; Delivered in a long-term care facility.	Intervention was delivered by a certified mindfulness trainer.
Tsujishita *et al*., 2020	VR-based exposure therapy	The participants viewed a VR environment in which they had previously fallen, while performing relaxation training (via heartbeat biofeedback).	2 × 10-minute bouts of exposure, delivered within a single session.	Delivered face-to-face; Researcher; 1–1; Delivered in the research facility.	Data not reported
Van Haastregt *et al*., 2007; Zijlstra *et al*., 2009, 2011	AMB-CBT	The intervention used the AMB, developed by Tennstedt *et al*. (1998) and translated it into Dutch. Please see the Tennstedt *et al*. (1998) entry in this table for full description of the intervention. There were two key additions: An extra booster session was added 6 months after the final session to help overcome the reduction in intervention effect that was observed in the Tennstedt *et al*. (1998) study. The weekly frequency changed from twice a week to once a week to allow the participants to consolidate and integrate the intervention elements into their daily activities.	8 weekly 2-hour sessions; plus a booster session 6 months after final session.	Delivered face-to-face; Geriatric nurses; group sessions (average 10 participants per group); Delivered in local community centres.	Data not reported
Wetherell *et al*., 2016	CBT	Activity, balance, learning and exposure (ABLE) intervention: an integration of a fall prevention exercise programme with exposure-based CBT. Within these sessions the following topics were covered: education about anxiety; identifying avoidance behaviours; developing an understanding of individual triggers and avoidance behaviours related to CaF; conducting different exposure practices; cognitive restructuring to help change negative thoughts to positive ones.	8 weekly 60-minute sessions.	Delivered face-to-face; Physiotherapist (who had supervision from a psychologist); 1–1 sessions, delivered in the participants’ homes.	The intervention was supervised by a licensed psychologist.
Wetherell *et al*., 2018	CBT	The same intervention from Wetherell *et al*., 2016 is used, but delivered to a new group of participants.	8 weekly 60-minute sessions.	Delivered face-to-face; Physiotherapist (who had supervision from a psychologist); 1–1 sessions; Delivered in the participants’ homes.	The intervention was supervised by a licensed psychologist.
Zijlstra *et al*., 2013	AMB-CBT	The same intervention (Dutch version of AMB) as van Haastregt *et al*., 2007; Zijlstra *et al*., 2009; Zijlstra *et al*., 2011; but delivered to a new group of participants.	8 weekly 2-hour sessions, plus booster session 2 months after the final session.	Delivered face-to-face; Geriatric nurses; group sessions (average 10 participants); Delivered in local community centres.	Data not reported

AMB = A Matter of Balance; CaF = Concerns about Falling; CBT = Cognitive Behavioural Therapy; Short FES-I = Short Falls Efficacy Scale – International; VR = Virtual Reality.

We also collated information pertaining to feasibility and acceptability of the individual interventions. Specifically, we extracted number and length of sessions as markers of feasibility, while adherence rates and recruitment rates (the percentage of participants enrolled compared to those invited) for each intervention were extracted as markers of acceptability. Additional analyses (e.g. pre-to-post t-tests) were conducted where papers provided means and standard deviations without key statistical results. Controlled trials (both RCTs and non-randomised trials) were classified as efficacious if they reported a statistically significant improvement in CaF (or balance confidence) at any point post intervention (compared to either pre-intervention and/or to a control group post-intervention; see Table 3).

**Table 3:** A table showing a breakdown of the efficacy of randomised and non-randomised controlled trials.

Psychological Intervention	Number of interventions using method	Median number of sessions across interventions	Average session length (mins) across interventions	Study ^a^
**Efficacious interventions**				
**RCT**				
CBT	6	8	49.5	†* [64]; [56]; [20, 44]; [63]
				† [41–43]
				* [67]
AMB-CBT	3	8	96	†* [48–50]
				† [19, 39, 40]; [32]
Motivational interviewing	2	4.5	25	† [60]; [72]
VR-based exposure	1	12	40	† [62]
Guided relaxation and imagery	1	12	15	† [70]
**Non-randomised controlled trial**				
Behavioural therapy	1	10	30	†* [55]
Mindfulness	1	8	100	†* [54]
**Non- Efficacious**				
**RCT**				
CBT	1	~19 guided exposure; ~3.5 cognitive restructuring	Not mentioned	[45, 46, 47]
Generalised CBT	1	6	10	[71]
Psychological education	1	11	120	[36]
Relaxation training and falls-based education	1	52	60	[37]
Visual feedback and cognitive reframing	1	Not mentioned	Not mentioned	[68]
Motivational interviewing	1	8	22.5	[66]
Motor imagery training	1	18	20	[58]
Hypnosis	1	4	30	[59]

^*^a^*^ *Studies from the same intervention (*e.g. *efficacy and then subsequent cost-benefit analysis) are group together within a single square bracket;*

^*^ *significant improvement in CaF compared to baseline;*

^*^†^*^ *Significant improvement in CaF, when compared to controls (at post-intervention); AMB = A Matter of Balance; CaF = Concerns about falling; CBT = Cognitive Behavioural Therapy; RCT = Randomised Controlled Trial; VR = Virtual Reality.*

## Results

### Study selection

**Figure 1:**
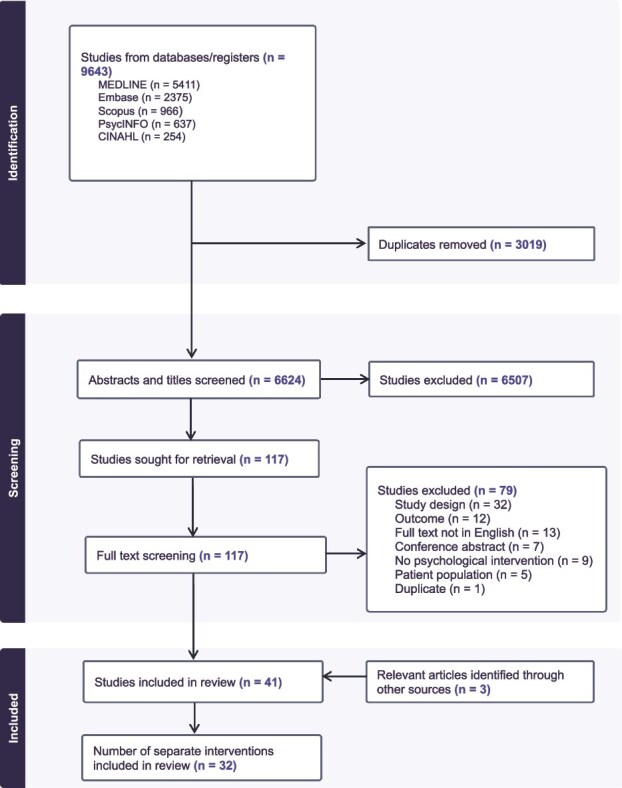
Full screening PRISMA flowchart. Notes: Articles were identified via other sources were found by searching reference lists of all other included studies.

The initial searches identified 9643 articles, of which 3019 duplicates were removed. The remaining 6624 articles underwent title and abstract screening, resulting in 117 full-texts being assessed for eligibility. Of these, 79 were excluded, leaving 41 papers for final inclusion—three of which were added through additional searches [36–38]. The full screening process can be seen in the PRISMA flowchart in Figure 1.

### Psychological informed interventions and categorisation

The 41 studies reported on 32 distinct interventions, with multiple studies often reporting different components of the same intervention (e.g. feasibility, acceptability, efficacy etc.). Studies reporting on the same intervention were grouped together for data extraction and analysis (these are: [19, 20, 39–50]).

### Study characteristics

There were four different study designs included in the review: 21 RCTs, five non-controlled trials, four case studies, and two non-randomised controlled trials. Across all studies, 3674 participants were included, with sample sizes ranging from a case study of one participant [51–53], to an RCT with 434 participants [32]. Participants were predominantly female (mean = 70.6%), with only one intervention having more males than females [54]. Mean ages ranged from 63.5 [55] to 87 years [53]. Most interventions targeted community-dwelling older adults (*n* = 23), while others recruited participants from long-term care facilities (*n* = 5) [46, 52, 54, 56, 57] or in-patient settings (*n* = 4) [20, 58–60]. Data were collected in various countries, most commonly United States (*n* = 11), the Netherlands (*n* = 5), Taiwan (*n* = 3), the United Kingdom (*n* = 3), Australia (*n* = 2) and Iran (*n* = 1).

### Intervention characteristics

The interventions were delivered by various different healthcare professional including: physiotherapists (*n* = 8) [20, 21, 46, 55, 60–63], nurses (*n* = 7) [19, 49, 56, 60, 64–66], researchers (*n* = 5) [53, 54, 58, 67, 68], psychologists (*n* = 3) [36, 52, 57], and others (e.g. healthcare assistants [43], audiologists [51], physicians [59], volunteer leaders [69] or unspecified facilitators [32, 38]). Three remaining studies were either self-administered [70], delivered online [71] or did not report who delivered the intervention [37].

Of the 17 interventions reporting training requirements, eight were delivered by practitioners who were already trained in the relevant psychologically-informed method [21, 36, 45, 51, 54, 59, 63, 70], while nine interventions provided additional training that ranged from 2 [19, 69, 72] to 5 days [43, 60, 66], with intervention delivery often supported by a clinical psychologist [19, 20, 43, 57, 60, 66, 67, 69, 72].

**Figure 2:**
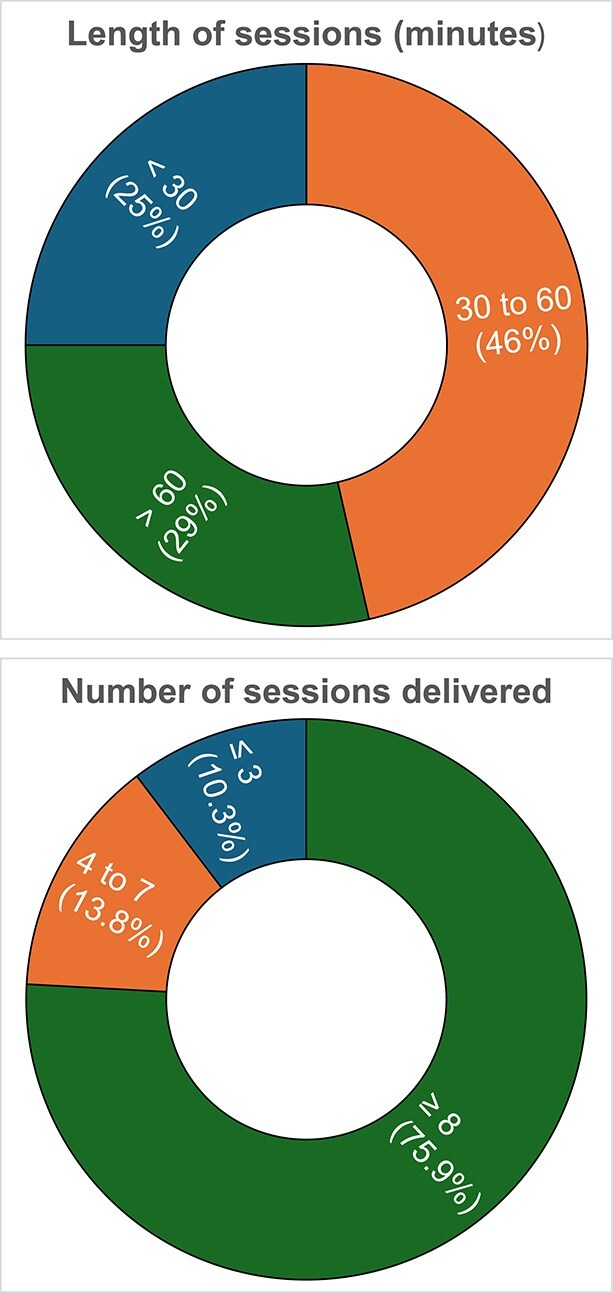
Pie charts detailing the number (top) and length of sessions (bottom) delivered in the included interventions (*n* = 32). Note: interventions that did not include details on their sessions delivered (*n* = 3) or their length (*n* = 3) are excluded from the pie charts.

When exploring markers for feasibility (number and length of sessions), the median number of sessions across the interventions was eight, with a median session length of 42.5 minutes (Figure 2). The shortest intervention—a single 20-minute session of motivational interviewing session delivered alongside usual in-hospital care—significantly reduced CaF [60]. Conversely, the longest intervention—weekly 60-minute psychoeducation and relaxation training sessions over 1 year—did not [37].

Adherence rates—one of our key markers of acceptability—varied depending on the authors’ criteria for ‘adherence’. For 21 interventions, successful adherence was defined as participants attending 100% of the sessions [21, 36, 38, 43, 46, 51–54, 56, 58–60, 63–66, 68, 70–72], of which nine studies reported 100% adherence, seven >90% adherence, and the other five ranging from 60% to 90% adherence. In two interventions, attendance of over 70% was required; both studies reported adherence in ~66% of participants [19, 49]. Four other interventions set the threshold at attending >60% of the sessions, and adherence rate in these interventions ranged from 63% to 89% [20, 32, 37, 69]. One intervention only required attendance of above 50% to qualify as adherence, and the adherence to this intervention was 77% [67].

Across the 32 interventions, 17 reported their recruitment rates (our other key acceptability metric). The median recruitment rate was 66%. Four interventions reported over 90% recruitment rate [36, 58, 65, 70]. Recruitment rates of between 70% to 90% were reported by three interventions [46, 56, 71], 50–70% reported by seven interventions [20, 43, 59, 60, 64, 66, 67], and four interventions reported recruitment rates of 50% or less [19, 49, 63, 72]. Interestingly, three studies with some of the lowest recruitment rates (between 39% and 53%) involved the delivery of motivational interviewing (either as a stand-alone intervention [60, 72] or in combination with CBT [19]).

There were no clear associations between intervention duration, adherence and efficacy—nor any clear differences between these outcomes based on the setting in which interventions were delivered. It should, however, be noted that interventions delivered within hospital settings tended to consist of more uncommon methods (e.g. hypnosis [59], motor imagery [58] and motivational interviewing [60]), rather than CBT, which was commonly delivered in community and long-term care settings.

Of the 21 RCTs, 14 used a CaF assessment tool [19, 20, 36, 37, 43, 45, 49, 60, 62, 63, 66–68, 70], three used a balance confidence tool [32, 58, 72] and four used both CaF and balance confidence tools [56, 59, 64, 71]. Eleven out of 18 studies reported a significant reduction in CaF at follow-up [19, 20, 42, 50, 56, 60, 62–64, 67, 70]. Two interventions showed a significant improvement in balance confidence at follow-up [32, 72] (Table 3). Among the eight non-efficacious RCTs, one used generalised CBT rather than a CaF-specific protocol [71], while another utilised an unstructured psychological intervention whereby physiotherapists applied personalised psychologically informed strategies based on their own clinical reasoning [46]. Interestingly, one intervention delivered with caregivers present was also ineffective at reducing CaF [36]. There were five non-controlled trials that utilised a pre-post design. These consisted of four CBT interventions (two CBT and two structured ‘A Matter of Balance’ CBT programmes (AMB-CBT)) and one VR-based exposure, and all showed a significant post-intervention reduction in CaF (or an increase in balance confidence [69]). There were also four case studies, consisting of: cognitive physical therapy (*n* = 1), exposure therapy (*n* = 1), VR-based exposure (*n* = 1) and guided imagery (*n* = 1). All case-study interventions reported a reduction in CaF [51, 52] or increase in balance confidence [38, 53]; however no formal statistics were run due to their study design.

### Commonly used psychologically informed methods

Thirteen different psychologically informed techniques were used across the included studies. The most common approaches are described below.

#### Cognitive behavioural therapy

Fifteen interventions used a CaF specific CBT protocol [19–21, 32, 43, 45, 49, 52, 56, 57, 63–65, 67, 69], all of which significantly reduced CaF, except for one [45]. Five of these interventions [19, 32, 49, 65, 69] used the structured ‘A Matter of Balance’ CBT programme (AMB-CBT) [32], which combines cognitive restructuring, problem-solving and physical exercises to reframe unhelpful thoughts and improve balance confidence. Seven others utilised novel CBT protocols developed for that specific intervention, incorporating elements such as graded exposure, restructuring misconceptions about CaF, while promoting the belief that preventing a fall is controllable.

Across the 15 CBT interventions, there were 17 CBT intervention groups (two studies included and compared CBT only versus CBT with additional exercise [56, 64]). Of these, five delivered CBT only [43, 52, 56, 57, 64], seven delivered CBT sessions alongside exercise sessions in the same week [20, 21, 45, 56, 63, 64, 67]. Seven interventions delivered CBT and exercise within the same session [19, 20, 32, 45, 49, 65, 69]. Five of these latter interventions used the AMB-CBT protocol [19, 32, 49, 65, 69]. Most CBT interventions were delivered across eight sessions, with a median session length of 60 minutes. When comparing the CBT and CBT-AMB interventions, the CBT-AMB interventions had a median of 8.3 sessions compared to 9 for the other CBT interventions. On average, sessions of CBT-AMB lasted 108 minutes, while sessions of other CBT interventions lasted on average 50 minutes. Interestingly, the only non-efficacious CBT intervention was substantially longer than all others (average of ~30 sessions), and without a standardised protocol, relying instead on personalised CBT-based strategies. One other intervention used an online, generalised CBT intervention (i.e. not designed specifically to address CaF) [71]. No positive changes on CaF were observed following this intervention.

#### Exposure therapy

Four studies used exposure therapy, including three virtual reality (VR)-based protocols [53, 61, 62], and one traditional (i.e. ‘real life’) graded exposure approach [52]. VR-based interventions exposed participants to immersive threatening environments (e.g. a virtual reconstruction of an environment in which they fell) while seated, either passively [53] or using a computer mouse to navigate the environment [62] or while performing a balance task (walking on a treadmill) [61]). Interventions lasted between 2 to 12 sessions consisting of between 10 and 40 minutes, and all VR-based interventions reduced CaF. The traditional (i.e. ‘real life’) graded exposure therapy approach, delivered by a trainee clinical psychologist over 14 sessions, gradually exposed the participant to situations that evoked CaF [52], leading to large improvements in CaF.

#### Motivational interviewing

Three interventions exclusively used motivational interviewing [60, 66, 72], a conversational, patient-centred approach to enhance their intrinsic motivation towards behavioural change [73]. The shortest intervention—a single in-hospital session of ~21 minutes [60]—significantly reduced CaF immediately after the intervention (compared to the usual care control group). However, when the same team delivered a longer (8 sessions lasting 15–30 minutes) motivational interviewing intervention to community-dwelling older adults, no significant change in FES-I scores was observed [74], potentially due to lower baseline CaF levels. Another longer-term (8 weekly 30-minute sessions) motivational interviewing intervention delivered to older people after hip fracture led to modest improvements in balance confidence. All interventions were delivered by healthcare professionals with specialist training in motivational interviewing, ranging from a 2-day workshop [72] to ≥40 hours training [60, 74]. Additionally, two CBT-based interventions also incorporated motivational interviewing alongside other cognitive and behavioural strategies [45, 75].

#### Guided imagery

Two interventions used guided imagery [38, 70]—a technique that combines mental visualisation of scenarios (often those perceived as threatening) with relaxation techniques and awareness of bodily sensations [76]. Both interventions led to a significant reduction in CaF. One intervention [38] involved six weekly 35–45 minute group sessions (and an additional self-administered home programme). Participants were guided through relaxation and breathing techniques, followed by audiotaped instructions to visualise walking in their neighbourhood while focusing on changes in their physiological responses (e.g. breathing or muscle tension). The other intervention [70] was self-administered, with participants listening to a 15-minute audio-recordings twice a week for six weeks. The recording included deep breathing and relaxation exercises, progressing from visualising safe tasks (e.g. moving within the house) to more threatening scenarios (e.g. walking across an icy road).

#### Other techniques

Other less-common psychological techniques included cognitive physical therapy (*n* = 1) [51], behavioural therapy (*n* = 1) [55], hypnosis (*n* = 1), mindfulness (*n* = 1); motor imagery (*n* = 1), psycho-education (*n* = 1) [36], relaxation training (*n* = 1) [37], and combined visual physio-feedback and cognitive restructuring (*n* = 1) [68]. As illustrated in Table 3, the effects of these other techniques on CaF were mixed.

## Discussion

This scoping review identified 13 distinct psychologically informed interventions for addressing CaF in older adults. Given the high prevalence of CaF [1, 4] and its negative outcomes (e.g. increased falls and reduced quality of life [1, 8–10]), it is important for clinicians to have access to effective psychological techniques to address it. Among the 32 interventions (across the 41 included studies), CBT was the most common approach. This was used in 15 interventions, with significant improvements in CaF reported in all but one. The prominence of CBT aligns with its broader application in the clinical management of anxiety disorders [77]. Other techniques included exposure therapy (*n* = 4 interventions), motivational interviewing (*n* = 3) and guided imagery (*n* = 2), while less common methods included mindfulness (*n* = 1), hypnosis (*n* = 1), motor imagery (*n* = 1) and relaxation (*n* = 1).

The median adherence rate across the interventions was 93%, while the median recruitment rate was 66%, suggesting that the interventions were broadly acceptable to older adults. However, adherence rates varied from 60% [19] to 100% [20, 36–38, 41, 43, 47, 56, 61]), and most studies focused on objective measures (e.g. number of sessions completed) without asking participants directly about subjective experiences. This is perhaps problematic, as frail older adults have expressed a preference for less formal ‘talking therapies’ over structured CBT for anxiety management [78]. Likewise, numerous studies reported recruitment rates of <50% [19, 49, 63, 72]—with some of the lowest recruitment rates observed in interventions delivering motivational interviewing (either as a stand-alone intervention [60, 72] or in combination with CBT [19]). These findings underscore the importance of tailoring interventions to older adults’ preferences, which could be achieved through co-design methodologies to enhance both acceptability and efficacy [79].

A recent survey of falls prevention practitioners identified time as the largest barrier to the effective clinical management of CaF [18]. On average, the interventions included in this review consisted of eight sessions, lasting 42.5 minutes (total average dose ~6 hours), with CBT interventions being the longest. Further examination of the differences between CBT and CBT-AMB interventions revealed that CBT-AMB sessions lasted on average twice as long (108 minutes) compared to other CBT interventions (50 minutes). These durations may be impractical for many falls prevention services, especially considering the extensive training required for non-specialists to deliver formal CBT, often also requiring ongoing psychologist support. By contrast, non-CBT interventions were shorter, averaging 31 minutes per session, suggesting that these may be more feasible for clinical integration. Motivational interviewing, which emphasises patient-centred communication to enhance intrinsic motivation, was effective even when delivered in a single ~20-minute session in a hospital setting alongside usual care [60]. Despite its short length, this intervention led to significant reductions in CaF, demonstrating its potential as a feasible, scalable approach.

This scoping review provides a broad overview of 32 interventions (across the 41 studies) using 13 distinct psychologically informed techniques to address CaF in older adults. By including diverse study designs and focusing on both feasibility and acceptability, it highlights practical considerations for integrating these interventions into clinical practice. Furthermore, interventions were delivered across different settings (community, long-term care and hospital). Notably, there were no clear differences between settings with respect to the acceptability, feasibility or efficacy of the interventions, suggesting that these approaches may be equally applicable across clinical contexts. However, interventions delivered in hospital tended to comprise of more uncommon psychological strategies (e.g. hypnosis [59], motor imagery [58], and motivational interviewing [60]). Future work is therefore needed to better understand how to best implement more traditional psychological strategies within hospital settings.

Some limitations of this work should be acknowledged. Most studies focused on quantitative outcomes only, limiting insights into participants’ subjective experiences and acceptability (as well as the perceptions of the healthcare providers delivering the interventions). Uneven geographic representation, with a predominance of studies from high-income countries and interventions including mostly females, may also limit generalisability. Finally, inconsistencies in reporting intervention components and adherence hinder direct comparisons across studies.

From a practical perspective, falls prevention services may consider a stepped-care approach, starting with shorter interventions like motivational interviewing and escalating to more intensive approaches (e.g. CBT) for those requiring additional support. Future work should prioritise understanding the feasibility of implementing psychologically informed practices within falls prevention services by exploring the perspectives of clinical practitioners and older adults. Co-design approaches and implementation research could provide valuable insights into how psychological interventions can be adapted for real-world use in falls prevention services.

## Conclusion

This review mapped the existing psychologically informed interventions for managing CaF in older adults. Of the 32 interventions (across the 41 studies) reviewed, 13 different psychologically informed methods were identified, with CBT being the most common (used in 15 interventions). This was followed by exposure therapy (4 interventions) and motivational interviewing (3 interventions). Most interventions had a positive impact on CaF, but the time-intensive nature of many interventions and the additional training required for delivery raise questions about their feasibility in clinical practice. Future research should focus on identifying more time-efficient, acceptable approaches through collaboration with both clinicians and older adults to ensure successful implementation.

## Supplementary Material

A_A_Supplementary_materials_Final_Version_published_afaf281
